# Human papillomavirus DNA detected in fingertip, oral and bathroom samples from unvaccinated adolescent girls in Tanzania

**DOI:** 10.1136/sextrans-2018-053756

**Published:** 2019-01-13

**Authors:** Catherine F Houlihan, Kathy Baisley, Ignacio G Bravo, Miguel A Pavón, John Changalucha, Saidi Kapiga, Silvia De Sanjosé, David A Ross, Richard J Hayes, Deborah Watson-Jones

**Affiliations:** 1 Division of Infection and Immunity, University College London, London, UK; 2 Department of Clinical Research, London School of Hygiene and Tropical Medicine, London, UK; 3 MRC Tropical Epidemiology Group, London School of Hygiene and Tropical Medicine, London, UK; 4 French National Center for Scientific Research (CNRS), Laboratory MIVEGEC (CNRS IRD Uni Montp), Montpellier, France; 5 Infections and Cancer Laboratory, Cancer Epidemiology Research Program, Catalan Institute of Oncology (ICO), Barcelona, Spain; 6 CIBER-ONC, Hospitalet de Llobregat, Barcelona, Spain; 7 National Institute for Medical Research, Mwanza, Tanzania; 8 Mwanza Interventional Trials Unit, Mwanza, Tanzania; 9 Sexual and Reproductive Health, PATH, Seattle, Washington, USA

**Keywords:** adolescent, africa, epidemiology (general), hpv

## Abstract

**Objective:**

Human papillomavirus (HPV) DNA has been detected in vaginal samples from adolescent girls who report no previous sex and, in high-income settings, from fingertips, raising the possibility of non-sexual transmission. No such studies originate from East Africa which bears among the highest cervical cancer incidence and HPV prevalence worldwide. HPV-related oral cancer incidence is increasing, but oral HPV prevalence data from East Africa are limited. We aimed to describe the HPV DNA prevalence in genital and non-genital sites and in the bathroom of unvaccinated adolescent girls, and examine genotype concordance between sites.

**Methods:**

We nested a cross-sectional study of HPV in genital and extragenital sites within a cohort study of vaginal HPV acquisition. Unvaccinated girls age 16–18 years in Tanzania, who reported ever having had sex, were consented, enrolled and tested for the presence of HPV DNA in vaginal samples collected using self-administered swabs, oral samples collected using an oral rinse, and on fingertips and bathroom surfaces collected using a cytobrush.

**Results:**

Overall, 65 girls were enrolled and 23 (35%, 95% CI 23% to 47%) had detectable vaginal HPV. Adequate (β-globin positive) samples were collected from 36 girls’ fingertips and HPV was detected in 7 (19%, 95% CI 6% to 33%). 63 girls provided adequate oral samples, 4 (6%, 95% CI 0% to 13%) of which had HPV DNA detected. In bathroom samples from 58 girls, 4 (7%, 95% CI 0% to 14%) had detectable HPV DNA. Of the 11 girls with extragenital HPV, six had the same genotype in >1 site.

**Conclusion:**

We found a high prevalence of HPV in non-genital sites in adolescent girls and in their bathrooms, in this region with a high cervical cancer incidence. Concordance of genotypes between sites supports the possibility of autoinoculation.

## Introduction

Anogenital infection by human papillomaviruses (HPVs) are possibly the most common STI worldwide. Acquisition of cervicovaginal HPV occurs rapidly after first sex^1 2^ but has also been shown in adolescent girls and young women who report never having had sex.^3–5^ Limited studies have documented the presence of HPV DNA on inert surfaces^6 7^ and on fingertips,^4 8^ and suggested the possibility of transmission through non-penetrative sexual practices, including kissing.^9^ Anogential HPV detection has also been associated with practices that involve cleansing inside the vagina with cloths or fingers.^3 10^ It is unclear how important these observations are in explaining the high HPV prevalence and incidence in young women in East Africa which bears the highest incidence of cervical cancer and among the highest incidence and prevalence of HPV globally.^11 12^ Vaccination is most effective if delivered prior to first acquisition of HPVs. This is assumed to occur at first sex, but it is essential to determine whether there is potential for HPV transmission via fomites, non-penetrative sexual activity or intravaginal cleansing practices including douching, as infection through any of these routes could precede sexual debut.

Infection of the oral mucosa by oncogenic HPVs, particularly HPV16, has been strongly associated with some oropharyngeal cancers,^13^ a malignancy of increasing incidence in high-income settings and potentially under-reported in low-income regions including East Africa.^14^ Oral sex and vaginal HPV infections have been identified as risk factors for oral HPV DNA detection.^9 15^ However, few studies have examined risk factors for, and prevalence of, oral HPV infection in sub-Saharan Africa.^16^ In addition, with emerging evidence of the potential role of HPV in ocular cancers,^17^ understanding HPV carriage in non-genital sites is increasingly important.

The objective of our study was to describe HPV DNA prevalence in samples collected from the oropharynx, vagina, fingertips and bathrooms of adolescent girls in Tanzania who have had at least one sexual partner, to determine the genotype-specific HPV DNA prevalence in these sites and to highlight potential transmission mechanisms.

## Methods

### Cohort enrolment

A longitudinal study of HPV acquisition around the time of first sex enrolled 503 girls age 15 and 16 years in Mwanza, Tanzania, between January and August 2012. Study methods have been previously described.^3^ In summary, parental consent was required before participant assent. Girls were followed every 3 months for 18 months and provided one self-administered vaginal swab and face-to-face interview at every visit. Girls reporting sexual activity since the previous visit were offered pregnancy testing and were verbally screened for STI symptoms, which was provided according to national guidelines.^18^ HIV testing was offered at the final visit.

Girls who attended the final study visit between August 2013 and March 2014 were invited to participate in a cross-sectional study of HPV prevalence in fingertips, oral rinses and household swabs. For girls less than 18 years old, parental consent was required before participant assent; girls over 18 provided their own consent. A face-to-face questionnaire was administered to all study participants and collected information on household demographics and lavatory and bathing facilities.

### Sample collection

Vaginal samples were collected as part of the HPV Cohort Study, with a single self-administered Dacron-tipped swab observed by a trained nurse as described previously.^3^ Participants washed their hands with soap and water before and after vaginal sample collection. Nurses wearing clean gloves placed the swab into a non-sterile cryotube and stored samples in cool boxes with ice-packs in the field before daily submission to the laboratory in Mwanza for −20C storage.

Fingertip, oral and bathroom samples were collected on a separate visit (usually less than 1 week apart). For the fingertip samples, a study nurse wearing gloves rubbed a dry cervical cytology brush three times under each of the participant’s fingernails (including the thumb), then three times over the tip of each finger. The brush was dipped into a cryotube containing 1 mL of 0.1 M phosphate-buffered saline and agitated briefly before the procedure was repeated. Once sample collection was complete, the brush was placed into the saline solution and the handle cut with a pair of sterile disposable scissors so that the tube could be closed.

Oral samples were collected using a technique as described by the Centers for Disease Control.^19^ Briefly, 10 mL of mouthwash (Scope; Procter & Gamble) in a pre-prepared universal container was gargled for 5 s, then ‘swished’ around the mouth for 5 s, and the process was repeated for a total of 30 s.

Household samples were collected from the participant’s current residence, following signed informed consent from the head of household. Nurses collected one sample from the tap/bucket handle used by the participant for personal washing, and a second sample from the tap/bucket used by the participant before or after using the toilet. Nurses wore new gloves to collect every sample and used a cervical cytology brush since this method had been used in other studies of HPV detection on fomites.^6^ The brush was dipped into a universal container containing 0.5 mL of 0.1 M phosphate-buffered saline prior to being firmly wiped over the surface to be sampled. Fingertip, oral and household samples were stored in the field and submitted daily to the laboratory in the same way as vaginal samples, described above.

Samples were shipped to the Catalan Institute of Oncology, Barcelona, Spain and tested for the presence of viral DNA with the Linear Array HPV genotyping assay (Roche, USA). Using updated genotype nomenclature from the International Human Papillomavirus (HPV) Reference Center (www.hpvcenter.se), 36 HPV genotypes were tested for and classified as possibly/probably oncogenic (‘high risk’) (HPV16, 18, 31, 33, 35, 39,– 45,-51,–52,-56,–58,-59,–68), or undetermined oncogenic potential (“low risk”)(HPV6,-11,–26,-34,–40,-42,–44,-53,–54,-61,–62,-66,–67,-69,–70,-71,–72,-73,–81,-82,–83,−84, and −89).

All vaginal samples collected at the final visit were tested for HPV. Samples collected from fingertips, oral rinses and household bathrooms were tested if the participant had reported sex at any time during the study. The presence of human DNA β-globin was used to assess the quality of the sample from participants; fingertip, oral and vaginal samples that tested negative for β-globin were excluded. Household samples that were negative for β-globin were not excluded.

### Data management and statistical methods

Questionnaire data were double-entered into OpenClinica LLC (Akaza Research, MA, USA), and analysed using STATA V14.0 (StataCorp LP, Texas, USA). In samples from fingertips, oral rinses and self-administered vaginal swabs where β-globin was detected, the presence of any HPV genotype was tabulated against reported demographics, intravaginal practices and sexual behaviours. P values were calculated using Fisher’s exact test or chi-squared test. Study numbers were too small for multivariable analysis.

In households where more than one specimen was collected, results were combined at the analysis stage. Socioeconomic status was measured by combining data on ownership of common household items in the entire cohort (n=503) using principal component analysis.

## Results

### Demographics

Of 503 participants, all of whom were unvaccinated for HPV, enrolled in the cohort study, 416 (83%) attended the final 18-month study visit. Of these, 91 girls (22%) reported ever having had sex, of whom 69 (76%) consented to participate in the substudy and 22 (24%) girls (or their parents) refused consent. After consent, four girls did not provide any finger/oral/household samples and were excluded.

Of the 65 enrolled and eligible participants, 49 (75%) were under 18 years old (table 1). In total, 38 (58%) were not married and 44 (75%) lived in a household that accessed water from a shared well or bore hole. Most girls (N=47, 72%) reported having only one lifetime sexual partner, and reported having performed intravaginal cleansing (N=46, 71%). Of the 65 offered an HIV test at the final visit, 11 (17%) refused without explanation, 13 (20%) refused stating that they had recently tested negative and 41 (63%) accepted, of whom one tested positive (2%). Overall, 46 girls (71%) had vaginal HPV detected at least once during the 18 months of the cohort study.

**Table 1:** Human papillomavirus (HPV) prevalence in vaginal, fingertip, oral and household samples from 65 adolescent girls

	Overall	Vaginal HPV n (%)	Fingertip HPV n (%)	Oral HPV n (%)	Household HPV n (%)
HPV (at same visit)					
Vaginal HPV (adequate samples from all 65 girls)			p=0.19	p=0.62	p=0.62
Yes	23 (35)	n/a	4 (33)	2 (9)	2 (5)
No	42 (65)	n/a	3 (13)	2 (5)	2 (10)
Fingertip HPV (adequate samples from 36 girls*)		p=0.19		p=0.005	p=1.00
Yes	7 (19)	4 (57)	n/a	3 (43)	1 (17)
No	29 (81)	8 (28)	n/a	0 (0)	3 (12)
Oral HPV (adequate samples from 63 girls*)		p=0.62	p=0.005		p=0.12
Yes	4 (6)	2 (50)	3 (100)	n/a	1 (33)
No	59 (94)	2 (36)	4 (13)	n/a	3 (6)
Household HPV (samples from 58 girls)		p=0.62	p=1.00	p=0.20	
Yes	4 (7)	2 (50)	1 (25)	1 (25)	n/a
No	54 (93)	19 (35)	5 (19)	2 (4)	n/a
Demographics					
Age (years)		p=1.00	p=0.16	p=0.56	p=1.00
16–17	49 (75)	17 (35)	7 (26)	4 (8)	3 (7)
18–19	16 (25)	6 (37)	0 (0)	0 (0)	1 (8)
Residence		p=0.79	p=1.00	p=1.00	p=0.04
Urban	28 (43)	9 (32)	3 (17)	2 (7)	4 (15)
Rural	37 (57)	14 (38)	4 (22)	2 (6)	0 (0)
Married		p=0.44	p=0.07	p=0.03	p=0.63
Yes	38 (58)	8 (30)	5 (38)	4 (15)	1 (4)
No	27 (42)	15 (39)	2 (9)	0 (0.0)	3 (9)
Household wealth		p=0.95	p=0.003	p=0.04	p=0.08
High	17 (26)	6 (35)	1 (7)	0 (0)	1 (4)
Medium	21 (32)	8 (38)	0 (0)	0 (0)	0 (0)
Low	27 (42)	9 (33)	6 (55)	4 (16)	3 (20)
Sexual behaviour					
Recent vaginal sex†		p=0.47	p=0.17	p=0.09	p=0.46
Yes	10 (15)	2 (20)	3 (37)	2 (22)	1 (13)
No	55 (85)	21 (38)	4 (14)	2 (4)	3 (6)
Recent kissing (any type)†		p=1.00	p=1.00	p=0.63	p=0.63
Yes	25 (38)	9 (36)	2 (15)	2 (8)	2 (9)
No	40 (62)	14 (35)	5 (22)	2 (5)	2 (6)
Oral-penile contact ever		p=0.004	p=1.00	p=0.71	
Yes	5 (8)	5 (100)	1 (20)	0 (0)	0
No	60 (92)	18 (30)	6 (19)	4 (7)	0
Recent hand-genital contact with a male partner†		p=1.00	p=0.60	p=0.06	p=1.00
Yes	17 (26)	6 (35)	2 (29)	3 (18)	1 (6)
No	48 (74)	17 (35)	5 (17)	1 (2)	3 (7)
Intravaginal practices					
Recently cleansed inside the vagina†		p=0.79	p=0.40	p=0.63	p=0.62
Yes	40 (62)	15 (38)	5 (28)	2 (5)	2 (5)
No	25 (38)	8 (32)	2 (11)	2 (8)	2 (10)

P values are derived from Fisher’s exact test or χ^2^ tests.

*An adequate sample was one in which β-globin was detected.

†Recent sex/kissing or hand-genital contact is defined as reporting this in the period between the last visit of the cohort study attended and this visit.

n/a, not available.

### HPV results

All 65 participants provided a self-administered vaginal swab as part of the routine cohort study procedures, all of which had β-globin detected. Twenty-three (35%, 95% CI 23% to 47%) girls had vaginal HPV DNA detected (table 1).

Fingertip samples were provided by all 65 girls, of whom 29 were β-globin negative and were excluded (none of these samples had HPV DNA detected). Of the 36 girls with β-globin-positive fingertip samples, 7 (19%, 95% CI 6% to 33%) had HPV DNA detected. Oral samples were provided by 64 girls; all but one of which tested positive for β-globin. Four 4 (6%, 95% CI 0% to 13%) samples from these 63 girls had detectable HPV DNA. Household samples were collected from the bathrooms of 58 girls, 25 of whom had two separate samples collected. All samples were collected from water buckets/jugs used for personal washing or toileting; no samples were collected from taps since these did not feature in washing/toileting behaviours. HPV DNA was detected from the bathrooms of 4 (7%, 95% CI 0% to 14%) girls.

Fingertip HPV DNA and oral HPV DNA were detected more frequently in girls who were married (39% vs 9% in unmarried girls (p=0.07) for fingertip and 15% vs 0% (p=0.03) for oral), and in those with lower household wealth scores. Fingertip HPV DNA was found in 55% of girls from low wealth-index households versus 7% from high wealth-index households (p=0.003). Oral HPV DNA was detected in 16% of girls from lower wealth-index household compared with no girls in high-income households (p=0.04). Oral HPV was more commonly detected in girls who reported recent hand-genital contact (18% vs 2%, p=0.06). Household HPV was more common in urban households compared with rural (15% vs 0%, p=0.04).

Among the four girls with oral HPV, 2 (50%) had the same genotypes detected in a vaginal sample collected at the same time and three had the same genotypes on a fingertip sample (table 2). Among seven girls with fingertip HPV DNA, four (57%) had the same genotypes detected in their vaginal samples and three (43%) had the same genotypes detected in their oral sample (table 2).

**Table 2:** Viral genotypes in girls who had human papillomaviruses (HPVs) detected in any of fingertip, oral or household bathroom samples

Participant	Vaginal HPV types	Fingertip HPV types	Oral HPV types	Household HPV types
1	None	66, 84	None	None
2	None	**16, 44, 81, 89**	**16**	**16, 44, 81, 89**
3	51, 83	None	None	6, 16
4	None	54, 81	None	None
5	None	None	None	51, 18
6	**6, 52**, 59, 66	None	None	**6, 52**, 53
7	**81**	73, **81**	**81**	*
8	55, **66**	**66**	None	None
9	None	†	45	None
10	56, **89**	6, **89**	None	None
11	62, **68**	**68**	**68**	None

HPV genotypes in bold were detected in more than one site in the same individual.

*β-globin not detected; therefore, this sample was considered inadequate.

†No household sample was provided.

Four girls who had HPV detected in bathroom samples: one had the same genotype detected on a fingertip sample, another had a matching genotype on an oral sample and a third had the same genotype detected on a vaginal sample (table 2).

**Figure 1:**
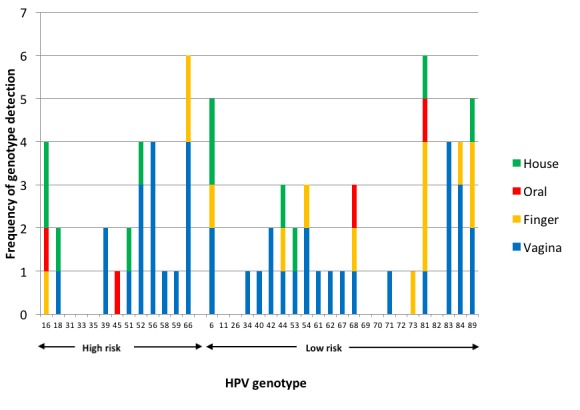
Human papillomaviruses (HPVs) detected in vaginal, oral, fingertip and household samples.

In vaginal samples, the most common *high-risk* HPVs were HPV56 and 66 (n=4 girls for each) (figure 1). No vaginal samples had HPV16 detected. The most common *low-risk* HPVs in vaginal samples were HPV83 (n=4 girls) and HPV84 (n=3 girls). In oral samples, two *high-risk* HPVs, HPV16 and 45, were detected (n=1 for each type), and two *low-risk* HPVs (HPV68, 81) were found. On fingertips, *high-risk* HPVs detected were HPV16 and 66. Among 17 *low-risk* HPVs detected, HPV81 was the most common (n=3 girls).

## Discussion

This is the first study to examine the overall HPV DNA prevalence and genotype-specific concordance in multiple anatomical sites and on the toilet/bathroom surfaces of unvaccinated adolescent girls. In Tanzanian girls aged 16 to 19 years who reported previous sex, mucosal HPV DNA was common on fingertips and was detected in almost one in five girls. Of those who had HPV detected on fingertips, almost 60% had the same genotype detected in their vagina and 43% had the same genotype in their oral cavity.

Although concordant HPV genotypes were seen in different anatomical sites in our participants, it is not possible to identify whether the source in these different sites was attributable to autoinfection (eg, an individual contaminating their own fingertips or oral mucosa from their genitals), to infection in all sites from one sex partner or HPV DNA deposition from a sex partner without infection. Deposition is possible since HPV DNA has been detected in semen: the detection of HPV DNA in our study could therefore simply reflect semen-associated HPV DNA which does not then go on to cause infection.^20^ Active HPV replication on fingertips is also unlikely since HPVs within *Alphapapillomaviruses* (tested for in this study) predominantly infect mucosal surfaces, as opposed to HPVs which infect keratinised epithelia (eg, *Beta* and *Gammapapillomaviruses*). Evidence from the risk factor analysis in this study demonstrated that HPV DNA on fingertips was associated with the detection of HPV DNA in oral samples, lower household wealth and being married, but surprisingly was not associated with hand-genital contact with a male partner, or recent sex. This could be attributable to a lack of disclosure of sexual behaviours, or could imply contamination of fingers with the participant’s own vaginal or oral HPV.

The high prevalence of HPV DNA in fingertips has been seen in the USA in newly sexually active girls (29%, n=127),^4^ but was understandably lower than in UK women who had genital warts (38%, n=8).^8^ In the study in newly sexually active girls, 60% of those with finger and genital HPV DNA had the same HPVs in both sites and, on further sequencing, the variant was the same in 27 of 28.^4^ This may indicate the same source of infection or transmission between these two sites. Conversely in oral HPV, one previous study illustrated no genotype concordance between oral HPVs and vaginal HPVs in 577 pregnant women,^21^ and suggested either a lack of evidence for autoinfection or differing rates of clearance and persistence of infection in these two sites.

HPV DNA was detected in 6% of 63 adequate oral samples in our study. A recent systematic review reported that 4% of 3690 healthy adult women tested positive for mucosal HPVs in the oral cavity,^22^ with a higher prevalence lower income country settings. Presence of oral HPV DNA has been associated with a higher number of sex partners, and with open-mouthed kissing,^9^ and with the number of oral sex partners in both men and women.^23^ However, a recent meta-analysis found evidence that oral sex was a risk factor for oral HPV in men, but not in women.^24^ This may be explained by the higher HPV prevalence in the genital tract of women compared with men,^25^ leading to a higher rate of oral HPV acquisition in heterosexual men who perform oral sex, and is also consistent with evidence of more efficient female-to-male than male-to-female HPV transmission.^26^ Autoinfection was further postulated as the explanation for vaginal HPV being a risk factor for oral HPV in women in South Africa.^16^ The number of adequate samples in our study, as well as and the numbers reporting oral sex, were too small to allow adequate power to examine this association.

Our study confirms that HPVs can be detected on inert surfaces in a tropical setting. HPV DNA on surfaces has been described in a few studies in high-income countries. A study of toilet seats in 23 airports, located in 13 countries, found HPV DNA in 23% of 101 β-globin positive samples.^6^ Similarly, HPV DNA was detected on 12 of 18 (67%) surfaces in a genito-urinary clinic in the UK^7^ and in 32 of 179 (18%) surfaces in a gynaecology clinic in Switzerland.^27^ The latter two studies reported HPV prevalence in all samples, not only β-globin-positive samples. Since HPV virions are not necessarily cell-associated, we did not consider the presence of human DNA necessary to indicate adequate surface sampling and have presented HPV prevalence on surfaces irrespective of β-globin presence. Our study provides evidence of HPV DNA detection in private bathrooms in an area with one of the highest prevalences of HPVs in the world and where intravaginal cleansing practices are common^10^ which may predispose contamination of bathroom surfaces and personal washing utensils. We were not able to test for viral infectiousness and HPV DNA detection does not necessarily correspond to virions able to infect a new host. However, HPV16 pseudovirions are able to infect cells after desiccation, displaying approximately 100%, 50% and 30% infectivity after being subjected to room temperature conditions for 1, 3 and 7 days, respectively.^28^ The carriage of HPV DNA on fingertips and on bathroom surfaces, which we have demonstrated, and the evidence that this virus can maintain infectiousness, supports the theoretical potential for the transmission of HPV infection during intravaginal cleansing. We have previously demonstrated evidence of the association between intravaginal cleansing and vaginal HPV DNA detection in this study population.^2^ However, we found no association between reporting intravaginal cleansing and fingertip HPV in this substudy.

HPV16, 18, 52 and 31 are the most prevalent genotypes in cervical specimens worldwide.^12^ In our study, the number of young sexually active girls was small, but HPV56, 68 and 83 were the most common genotypes overall, detected in 6% of vaginal specimens. Multiple genotype infections were seen in our study, which is also common. Interestingly, while HPV16 is the most common genotype in genital infections worldwide, and is responsible for most cases of cervical cancer,^29^ it was not detected in the vaginal samples from the cohort of girls presented here, but was present in oral, household and fingertip samples.

Limitations in our study include the small number of samples, particularly valid β-globin-positive samples from oral and fingertip samples, and the restricted power for the determination of associations between reported behaviours and the detection of HPV DNA at various sites. These small numbers mean that it was not possible to adjust for confounders of observed associations. Under-reporting of sexual behaviours has been described in the study region and is common in adolescent girls.^30^ This may have limited our ability to detect associations between the presence of HPVs and practices such as oral sex. However, our study participants had seen the study nurses every 3 months for the previous 18 months and developed a good relationship with them, potentially reducing this bias.

We report a high prevalence of HPV DNA in oral washes and fingertip samples in unvaccinated adolescent girls, as well as in their bathrooms. The study was carried out in a region where intravaginal cleansing is common, and there is an extremely high incidence of cervical cancer, as well as increasing incidence of HPV-related oropharyngeal cancer. Our data show that the concordance of genotypes between sites, particularly finger and oral HPV, was common, supporting the possibility of autoinoculation.

**Key messages**

- This is the first study to examine human papillomavirus (HPV) prevalence and genotype concordance in vaginal, oral and toilet/bathroom surfaces of adolescent Tanzanian girls.
- In sexually active girls aged 16–19 years, HPV was detected on fingertips in almost one in five, and in 6% of oral samples.
- We show that the concordance of genotypes between sites, particularly finger and oral HPV, was common, supporting the possibility of autoinoculation.
